# Infection Control Knowledge, Job Stress, and Self-Efficacy in Relation to Infection Control Performance Among Intensive Care Unit Registered Nurses: A Descriptive Cross-Sectional Study

**DOI:** 10.3390/healthcare14203443

**Published:** 2026-10-14

**Authors:** Ji Soo Lee, Eun Young Jeong

**Affiliations:** 1 Department of Nursing, Graduate School, Wonkwang University, Iksan 54538, Republic of Korea; 2 College of Nursing, Wonkwang University, Iksan 54538, Republic of Korea

**Keywords:** registered nurse, intensive care unit, infection control knowledge, infection control job stress, self-efficacy, infection control performance, healthcare-associated infection

## Abstract

Background/Objectives: Healthcare-associated infections remain a critical patient-safety concern in intensive care units (ICUs), where invasive procedures and immunocompromised patients elevate infection risk. Infection control knowledge, job stress, and self-efficacy have each been linked to infection control performance, but few studies have examined these factors simultaneously among ICU registered nurses (RNs). This study aimed to investigate ICU RNs’ infection control knowledge, infection control job stress, self-efficacy, and infection control performance, and to identify factors associated with infection control performance. Methods: This descriptive cross-sectional study surveyed 191 ICU RNs (of 219 recruited) with ≥6 months of ICU experience at two tertiary hospitals in the Republic of Korea (December 2024–January 2025) using structured, validated questionnaires. Data were analyzed with descriptive statistics, independent *t*-tests, one-way ANOVA (Scheffé/Games–Howell post hoc), Pearson correlation, and hierarchical multiple regression (SPSS 22.0). Results: Mean scores were 19.16 ± 1.07 (of 20) for infection control knowledge, 3.23 ± 0.67 (of 5) for infection control job stress, 3.62 ± 0.53 (of 5) for self-efficacy, and 4.82 ± 0.26 (of 5) for infection control performance. Infection control performance was positively correlated with knowledge (r = 0.151, *p* = 0.037), job stress (r = 0.161, *p* = 0.026), and self-efficacy (r = 0.178, *p* = 0.014). Hierarchical regression showed that current ICU department (surgical, cardiac, emergency, trauma, and general ICU vs. internal medicine ICU as reference), infection control job stress (β = 0.188, *p* = 0.008), and self-efficacy (β = 0.157, *p* = 0.022) significantly predicted infection control performance, together explaining 17.4% of the variance (F = 5.995, *p* < 0.001); infection control knowledge was a nominally significant predictor in this model (β = 0.164, *p* = 0.016), but this association was not robust to sensitivity analyses (Spearman ρ = 0.034, *p* = 0.636; heteroscedasticity-robust regression *p* = 0.095) or to a covariate-adjusted robustness check (*p* = 0.091), and should be interpreted with caution. Conclusions: Infection control job stress, self-efficacy, and current department were robustly associated with infection control performance among ICU RNs across all sensitivity analyses; infection control knowledge showed a comparatively weaker, fragile association that did not survive rank-based, heteroscedasticity-robust, or covariate-adjusted sensitivity checks. Effect sizes were small to moderate overall, and the final model explained only 17.4% of variance. Department-tailored education and organizational support strategies—channeling job stress constructively and strengthening self-efficacy through hands-on training and mentoring—may help sustain and improve infection control performance; recommendations regarding knowledge-related training should be treated as preliminary given the fragility of this association.

## 1. Introduction

Healthcare-associated infections (HAIs) remain a critical patient-safety concern worldwide, intensified by the recurring emergence of infectious diseases such as severe acute respiratory syndrome (SARS), Ebola virus disease, Middle East respiratory syndrome (MERS), and coronavirus disease 2019 (COVID-19); HAIs increase morbidity and mortality and prolong hospital stay and cost [1,2]. Intensive care units (ICUs), characterized by frequent use of invasive devices and a high proportion of immunocompromised patients, have markedly higher HAI rates than general wards. A landmark multinational point-prevalence study across 1265 ICUs in 75 countries found that more than half of ICU patients had a confirmed or suspected infection on the study day [3], and the World Health Organization similarly estimates that approximately 30% of ICU patients globally are exposed to a healthcare-associated infection [4]. In the Republic of Korea specifically, 5348 HAIs were reported across 372 ICUs over a single year according to national surveillance data [5].

ICU registered nurses (RNs) have the most frequent direct patient contact among healthcare providers and are therefore central to translating infection control guidelines into everyday practice [6]. Infection control performance is shaped not only by individual competence but also by workload, organizational environment, and psychological factors. Infection control knowledge has been identified as a core determinant of performance [7], although some studies report no significant association between the two [8], leaving the relationship unsettled. ICU RNs also routinely experience infection-control-specific job stress arising from high-risk procedures, isolation precautions, and prolonged personal protective equipment use [9]. Self-efficacy—an individual’s belief in their capacity to organize and execute the courses of action required to produce a given outcome [10]—has likewise been proposed as a key psychological resource for infection-prevention behavior; international evidence has linked nurses’ self-efficacy to their attitudes and practices around infection prevention, control, and antibiotic stewardship [11]. However, few studies have examined infection control knowledge, job stress, and self-efficacy simultaneously as predictors of infection control performance specifically among ICU RNs. This study therefore aimed to (1) describe the general characteristics of ICU RNs; (2) determine their levels of infection control knowledge, infection control job stress, self-efficacy, and infection control performance; (3) compare these variables across general characteristics; (4) examine the correlations among these variables; and (5) identify factors associated with infection control performance, in order to inform education and organizational strategies that support sustained infection control performance in ICUs.

These three factors were selected for joint examination because they represent conceptually distinct but complementary determinants of infection control behavior: infection control knowledge reflects the cognitive/informational basis for correct practice, infection control job stress reflects a proximal environmental-psychological demand that may either impair or, per activation-based models of stress, sharpen vigilant performance, and self-efficacy reflects the motivational-volitional belief that translates knowledge into sustained action even under demanding conditions. Prior studies have typically examined these constructs in isolation or in pairs, leaving unclear whether each retains independent explanatory value once the others are accounted for; a joint, hierarchical examination therefore provides a more conceptually complete test of their relative contributions to infection control performance.

## 2. Materials and Methods

### 2.1. Study Design

This study used a descriptive cross-sectional survey design to examine the levels of infection control knowledge, infection control job stress, self-efficacy, and infection control performance among ICU RNs, and to identify factors associated with infection control performance. This study followed the STROBE (Strengthening the Reporting of Observational Studies in Epidemiology) reporting guideline for cross-sectional studies; the completed checklist is provided as Supplementary Materials (Table S1).

### 2.2. Participants

Participants were ICU RNs, excluding RN managers, with ≥6 months of ICU experience who provided direct patient care at two tertiary hospitals in J Province, Republic of Korea. The required sample size, calculated with G*Power 3.1.9.7 (Heinrich Heine University Düsseldorf, Düsseldorf, Germany; α = 0.05, power = 0.90, effect size = 0.15, 16 predictors) based on a prior study [12], was 175; accounting for anticipated attrition, 219 RNs were recruited. Recruitment proceeded as a convenience sample of consenting volunteers approached through the participating nursing departments; the number of eligible nurses who were approached but declined, or who were not approached, was not centrally tracked, so a formal participation/response rate could not be calculated. After excluding 28 incomplete or insincere responses, 191 questionnaires were analyzed, exceeding the statistically required sample size. Incomplete responses (*n* = 25) were defined as questionnaires missing responses to 10% or more of items. Insincere responses (*n* = 3) were identified using two criteria defined a priori: (a) an invariant, straight-line response pattern across an entire multi-item Likert scale (e.g., selecting the same response option for all 32 job-stress items), and (b) a total completion time implausibly short given the 119-item instrument and an approximately 15 min mean completion time (Section 2.4).

### 2.3. Measures

A structured self-administered questionnaire (119 items) assessed general characteristics (13 items) and four main study variables. Permission to use all instruments was obtained from the original developers/adaptors by e-mail prior to data collection.

Infection Control Knowledge (20 items): the standard-precautions knowledge tool developed by Cho [13] for ICU and emergency-room RNs. Each item was scored 1 (correct) or 0 (incorrect); total scores range from 0 to 20, with higher scores indicating greater knowledge. Reliability was Cronbach’s α = 0.74 at development; in this study, Kuder–Richardson Formula 20 (KR-20), appropriate for dichotomously scored items, yielded a reliability coefficient of 0.35, indicating low internal consistency for this scale in the present sample; item-level statistics and further discussion of this finding are reported in Section 3.2, Section 3.6 and Section 4.

Infection Control Job Stress (32 items): the infection control nurses’ job stress tool developed by Heo [9] and adapted for infection control job stress by Jang [14], comprising four subdomains—quantitative workload (9 items), qualitative workload (11 items), organizational factors (6 items), and interpersonal conflict (6 items)—rated on a 5-point Likert scale and reverse-scored so that higher scores indicate greater job stress (original α = 0.96 [9]; adaptation α = 0.95 [14]; study α = 0.96).

Self-Efficacy (17 items): the General Self-Efficacy Scale developed by Sherer and Maddux [15] and adapted for Korean clinical RNs by Jung [16]; higher scores indicate greater self-efficacy (original α = 0.71 [15]; adaptation α = 0.94 [16]; study α = 0.95).

Infection Control Performance (37 items): the infection control performance tool developed by Kim [17] and revised by Hong and Park [18], covering six domains specified in national healthcare accreditation standards—hand hygiene, vascular catheter infection control, urinary tract infection control, pneumonia management, isolation, and disinfection/sterilization management; higher scores indicate greater performance (original α = 0.83 [17]; revision α = 0.95 [18]; study α = 0.95).

### 2.4. Data Collection

Data were collected from December 2024 to January 2025. The researcher explained the study purpose and procedures to the nursing departments of two tertiary hospitals in J Province and obtained departmental approval before recruiting participants. Structured questionnaires were distributed on-site to ICU RNs who provided written informed consent; completion took approximately 15 min. Completed questionnaires were sealed in individual envelopes and collected directly by the researcher on a subsequent visit.

### 2.5. Statistical Analysis

Data were analyzed using SPSS/WIN 22.0 (IBM Corp., Armonk, NY, USA). General characteristics and study variables were summarized with frequencies, percentages, means, and standard deviations. Group differences in infection control knowledge, job stress, self-efficacy, and performance by general characteristics were examined with independent *t*-tests and one-way ANOVA; Scheffé and Tukey post hoc tests were used, with Welch’s test and Games–Howell post hoc comparisons applied when the assumption of homogeneity of variance was violated. Relationships among the four main variables were examined with Pearson correlation coefficients. Factors associated with infection control performance were identified using hierarchical multiple regression: Model I entered general characteristics that differed significantly in univariate analyses, followed by infection control knowledge (Model II), infection control job stress (Model III), and self-efficacy (Model IV), with model fit assessed via the F-test, variance inflation factors (multicollinearity), and the Durbin–Watson statistic (residual independence).

Because the infection control knowledge and performance scores are bounded and preliminary inspection indicated pronounced ceiling effects, the distributional assumptions underlying the independent *t*-tests, one-way ANOVA, and Pearson correlations involving these two variables were examined directly: skewness, excess kurtosis, and the Shapiro–Wilk test were computed for both variables, and Spearman’s rank correlation was computed alongside Pearson’s r for all pairwise associations among the four main study variables as a sensitivity check. For the hierarchical regression model, in addition to the Durbin–Watson statistic and variance inflation factors, residual diagnostics were expanded to include the Shapiro–Wilk test for residual normality, the Breusch–Pagan test for heteroscedasticity, and leverage/Cook’s distance values to screen for unduly influential observations; heteroscedasticity-consistent (HC3) standard errors were additionally estimated for the final model as a sensitivity analysis. The order of variable entry in the hierarchical regression (department, then infection control knowledge, then infection control job stress, then self-efficacy) followed an a priori conceptual sequence from contextual/structural (department) to cognitive (knowledge) to environmental-psychological (job stress) to motivational-volitional (self-efficacy) factors, consistent with social-cognitive models in which self-efficacy is positioned as the most proximal determinant of sustained performance [10,19]. Full results of these supplementary analyses are reported in Section 3.6.

Because infection control job stress, self-efficacy, and infection control performance were all self-reported within the same questionnaire administration, common method bias (CMB) was a concern. To assess the potential risk of CMB due to the self-reported nature of the data, Harman’s single-factor test was performed on the raw item-level data. The results indicated that a single factor accounted for only 19.16% of the total variance, which is well below the 50% threshold conventionally used to infer that CMB is not a serious concern. Although this result provides some reassurance, Harman’s single-factor test is a relatively weak and conservative diagnostic; it cannot rule out CMB, and the possibility that CMB has inflated same-source associations—particularly the counterintuitive positive association between job stress and performance (Section 3.3, Section 3.4 and Section 3.5)—should be weighed explicitly when interpreting these findings (Section 4). Group differences in Section 3.3 were examined across multiple general characteristics without adjustment for multiple comparisons and should therefore be interpreted as exploratory and hypothesis-generating rather than confirmatory.

### 2.6. Ethical Considerations

This study was approved by the Institutional Review Board of Wonkwang University (approval number: WKUH IRB 2024-11-017-008; approved 9 December 2024). Prior to data collection, participants were fully informed of the study purpose and procedures, their right to voluntary participation and withdrawal without penalty, and the anonymity and confidentiality of their responses. Written informed consent was obtained from all participants. Collected data were used only for the stated research purpose, kept confidential, stored securely for three years, and will be destroyed thereafter.

## 3. Results

### 3.1. General Characteristics of Participants

Participants (*N* = 191) were predominantly female (84.3%), with a mean age of 29.73 ± 6.45 years; 61.8% were under 30 years old. Most were unmarried (75.9%) and held a bachelor’s degree (79.1%); 96.3% were staff RNs. Total clinical experience was most commonly <3 years (36.1%). The current ICU department was emergency ICU (25.7%), surgical ICU (23.0%), internal medicine ICU (17.3%), trauma ICU (15.7%), general ICU (10.5%), and cardiac ICU (7.9%). ICU experience was most commonly 1–<3 years (32.5%). Most participants (57.6%) had attended one infection control training session in the past 12 months, and 23.6% had attended none. Most participants (96.3%) worked a three-shift rotation. Other characteristics summarized in Table 1, including average daily patient load, did not differ significantly across the four main study variables (Section 3.3) or enter the final regression model (Section 3.5) and are therefore not discussed individually here.

### 3.2. Levels of Infection Control Knowledge, Job Stress, Self-Efficacy, and Performance

Infection control knowledge averaged 19.16 ± 1.07 (of 20; 95.8%). Infection control job stress averaged 3.23 ± 0.67 (of 5); subdomain scores were highest for quantitative workload (3.51 ± 0.81), followed by organizational factors (3.16 ± 0.80), interpersonal conflict (3.11 ± 0.76), and qualitative workload (3.09 ± 0.72). Self-efficacy averaged 3.62 ± 0.53 (of 5). Infection control performance averaged 4.82 ± 0.26 (of 5); subdomain scores were highest for pneumonia management (4.91 ± 0.25), followed by vascular catheter infection control (4.89 ± 0.26), disinfection and sterilization management (4.87 ± 0.31), isolation (4.87 ± 0.30), urinary tract infection control (4.80 ± 0.32), and hand hygiene (4.66 ± 0.40). Distributional screening confirmed pronounced ceiling effects for both bounded scores: infection control knowledge (skewness = −1.57, excess kurtosis = 3.37; Shapiro–Wilk W = 0.760, *p* < 0.001; 49.7% of participants scored the maximum 20/20) and infection control performance (skewness = −2.24, excess kurtosis = 5.17; Shapiro–Wilk W = 0.707, *p* < 0.001; 30.4% scored the scale maximum), confirming that the normality assumption for parametric comparisons involving these two variables was not satisfied and that the corresponding results (Section 3.3, Section 3.4 and Section 3.5) should be interpreted together with the sensitivity analyses in Section 3.6.

### 3.3. Differences in Study Variables by General Characteristics

Infection control knowledge differed significantly by total clinical experience (F = 2.85, *p* = 0.039), with RNs with <3 years of experience scoring highest.

Infection control job stress differed significantly by sex (t = −3.45, *p* = 0.001), age (t = −2.90, *p* = 0.004), marital status (t = −2.56, *p* = 0.011), education (F = 5.29, *p* = 0.006), total clinical experience (F = 4.87, *p* = 0.003), current department (F = 3.64, *p* = 0.004), and ICU experience (F = 2.71, *p* = 0.047). Job stress was higher among women, RNs ≥30 years, married RNs, those with a master’s degree or higher, those with ≥5 years of total clinical experience, general-ICU RNs, and those with ≥5 years of ICU experience.

Self-efficacy differed significantly by the number of infection control training sessions attended in the past 12 months (F = 3.22, *p* = 0.042), with RNs who attended ≥2 sessions scoring highest.

Infection control performance differed significantly by current department (F = 4.48, *p* = 0.001), with surgical, trauma, and general ICU RNs scoring higher than internal medicine ICU RNs. Effect sizes for all comparisons above ranged from small to medium (Cohen’s d = 0.43–0.69 for the sex/marital-status/age comparisons; η^2^ = 0.033–0.134 for the one-way ANOVA comparisons), and are reported with 95% confidence intervals in Section 3.6. Because ten univariate comparisons were tested here without correction for multiple comparisons, and several associated *p*-values were close to 0.05 (*p* = 0.039–0.047), these comparisons should be interpreted as exploratory and hypothesis-generating rather than confirmatory.

### 3.4. Correlations Among Study Variables

Correlations among the study variables are presented in Table 2. Infection control performance was positively correlated with infection control knowledge (r = 0.151, *p* = 0.037), infection control job stress (r = 0.161, *p* = 0.026), and self-efficacy (r = 0.178, *p* = 0.014). Self-efficacy was positively correlated with infection control knowledge (r = 0.161, *p* = 0.026) and negatively correlated with infection control job stress (r = −0.153, *p* = 0.035). Infection control job stress was negatively correlated with infection control knowledge (r = −0.159, *p* = 0.028). Because both infection control knowledge and infection control performance departed from normality (Section 3.2), Spearman’s rank correlation was computed alongside Pearson’s r as a sensitivity check for all six pairwise associations (Section 3.6). The Pearson and Spearman results converged for three of six pairs; notably, the correlation between knowledge and performance was not significant under the rank-based method (ρ = 0.034, *p* = 0.636, versus Pearson r = 0.151, *p* = 0.037), indicating that this particular association should be interpreted with caution.

### 3.5. Factors Associated with Infection Control Performance

Hierarchical multiple regression was performed to identify factors associated with infection control performance (Table 3). Model I entered current department (the only general characteristic significantly associated with performance); Models II–IV successively added infection control knowledge, infection control job stress, and self-efficacy. All four models were statistically significant (Model I: F = 5.742, *p* < 0.001; Model II: F = 5.851, *p* < 0.001; Model III: F = 5.947, *p* < 0.001; Model IV: F = 5.995, *p* < 0.001), with explanatory power increasing stepwise from 11.1% (Model I) to 13.3% (Model II), 15.4% (Model III), and 17.4% (Model IV; R^2^ = 0.209, Adjusted R^2^ = 0.174). Durbin–Watson statistics (1.956–1.972) indicated no residual autocorrelation, and all variance inflation factors were below 10, indicating no multicollinearity problem.

In the final model, compared with internal medicine ICU (reference), surgical (β = 0.398, *p* < 0.001), emergency (β = 0.341, *p* < 0.001), general (β = 0.327, *p* < 0.001), trauma (β = 0.318, *p* < 0.001), and cardiac (β = 0.173, *p* = 0.026) ICU RNs showed significantly higher infection control performance. Infection control job stress (β = 0.188, *p* = 0.008), infection control knowledge (β = 0.164, *p* = 0.016), and self-efficacy (β = 0.157, *p* = 0.022) were each significant independent predictors of infection control performance. Unstandardized coefficients (B) with 95% confidence intervals for the final model, expanded residual diagnostics (linearity, homoscedasticity, residual normality, and influential-case screening), and heteroscedasticity-robust (HC3) standard errors are reported in Section 3.6; under the HC3 specification, infection control knowledge and the cardiac-ICU department contrast no longer reached significance, while job stress and self-efficacy remained significant.

### 3.6. Additional Sensitivity and Diagnostic Analyses

The following supplementary analyses were conducted on the final analytic sample (*N* = 191) to evaluate the robustness of the primary findings reported in Section 3.2, Section 3.3, Section 3.4 and Section 3.5.

The correlation between knowledge and performance, significant under Pearson’s method, was not significant under the rank-based Spearman method (Table 4); the knowledge–self-efficacy correlation was similarly attenuated to a non-significant, borderline level. The associations of job stress and self-efficacy with performance were robust to this sensitivity check, whereas the job stress–self-efficacy correlation was attenuated to non-significance (ρ = −0.102, *p* = 0.161).

Residual diagnostics for this model indicated that, while the Durbin–Watson statistic (1.972) and all variance inflation factors (1.01–1.91) were within conventional acceptable ranges, the residuals departed from normality (Shapiro–Wilk W = 0.860, *p* < 0.001; skewness = −1.67, excess kurtosis = 3.65) and showed evidence of heteroscedasticity (Breusch–Pagan LM = 29.16, df = 8, *p* < 0.001). Leverage values exceeded the 2p/n screening threshold for 5 of 191 cases, and Cook’s distance exceeded the conventional 4/n threshold for 14 cases; none exceeded the more conservative threshold of 1.0, indicating no single case exerted an extreme, disproportionate influence. Under the HC3-robust specification (Table 5), infection control job stress and self-efficacy remained significant predictors, whereas infection control knowledge (*p* = 0.095) and the cardiac-ICU department contrast (*p* = 0.086) no longer reached significance—indicating that these two associations are less robust than the original OLS results suggest.

As shown in Table 6, Cohen’s d for the dichotomous comparisons ranged from 0.43 to 0.69 (small-to-medium); η^2^ for the ANOVA comparisons ranged from 0.033 to 0.134 (small, except department → performance, which was medium). Levene’s test indicated unequal variances only for the department → performance comparison (*p* = 0.025), for which Welch’s F is reported, consistent with Section 2.5; homogeneity was satisfied for all other comparisons (Levene’s *p* = 0.177–0.991). Given that ten univariate comparisons were tested without correction for multiple comparisons, and several *p*-values were close to 0.05 (*p* = 0.039–0.047), these findings should be treated as exploratory and hypothesis-generating.

Item-level accuracy (Table 7) ranged from 84.3% (item 4, concerning non-blood body fluids as an infection source) to 100% (item 10, unable to compute a correlation because it is answered correctly by all participants). Item-total point-biserial correlations were generally low (range −0.08 to 0.31), consistent with the low overall scale reliability (KR-20 = 0.35; Section 2.3); twelve of the 20 items showed a non-significant item-total correlation (*p* > 0.05), indicating that much of the item pool contributed little to differentiating participants’ knowledge levels in this high-scoring, homogeneous sample.

### 3.7. Covariate-Adjusted Robustness Check

We re-estimated the final regression model (Model IV, Section 3.5) with five additional, theoretically relevant covariates entered simultaneously regardless of their univariate significance: age (years), total clinical experience, ICU experience, number of infection control training sessions attended, and average daily patient load (workload). This robustness check (Model V) is a supplementary sensitivity analysis, not a re-specification of the primary a priori hierarchical model described in Section 2.5. For parsimony given the sample size, the three ordinal covariates (clinical experience, ICU experience, and training sessions) were entered as linear ordinal terms using the same category codings as Table 1, rather than fully dummy-coded; workload was entered as the binary indicator reported in Table 1 (≥3 vs. <3 patients/day), and age was entered continuously.

Model V remained statistically significant (R^2^ = 0.231, Adjusted R^2^ = 0.175, F(13, 177) = 4.100, *p* < 0.001) but explained essentially no additional variance beyond Model IV (ΔAdjusted R^2^ = 0.001); none of the five added covariates reached significance (all *p* ≥ 0.158), and all variance inflation factors remained below 4, indicating no problematic multicollinearity despite the expected correlation between age and clinical experience. Critically, the core findings were unchanged after this adjustment: all five department contrasts remained significant (under conventional estimation), infection control job stress (B = 0.078, *p* = 0.005; HC3-robust *p* = 0.004) and self-efficacy (B = 0.086, *p* = 0.014; HC3-robust *p* = 0.015) remained significant predictors under both conventional and heteroscedasticity-robust estimation, and infection control knowledge again showed the same fragile pattern reported in Section 3.6—nominally significant under conventional estimation (B = 0.041, *p* = 0.015) but not under HC3-robust standard errors (*p* = 0.091). This robustness check therefore reinforces, rather than alters, our interpretation that department, job stress, and self-efficacy are comparatively robust correlates of infection control performance, while the knowledge association remains fragile (Table 8).

## 4. Discussion

This study examined infection control knowledge, job stress, self-efficacy, and infection control performance among ICU RNs and identified factors associated with performance. Infection control knowledge was very high (95.8% of the maximum score), exceeding levels reported in prior studies using adapted versions of the same tool among clinical RNs (82.2–92.2%) [20], and was comparable to a recent study using the identical tool among ICU RNs (96.6%) [21]. This likely reflects strengthened infection control education and evaluation systems following the COVID-19 pandemic and healthcare accreditation reform [22]. Item-level analysis, however, showed the lowest accuracy (84.3%) on an item concerning non-blood body fluids as infection sources, versus 100% accuracy on a blood/mucous-membrane exposure item, suggesting that institutional training remains disproportionately focused on blood-borne exposure and should be broadened to cover non-blood body-fluid precautions more evenly [23]. These item-level results should be interpreted cautiously, however: the knowledge scale’s internal consistency in this sample was low (KR-20 = 0.35; Section 2.3 and Section 3.6), and the zero-order correlation between knowledge and infection control performance was not robust to a Spearman sensitivity analysis (Section 3.6); accordingly, the recommendation regarding non-blood body-fluid training is offered as a plausible, single-item-based hypothesis for future confirmation rather than an established finding. This pattern converges with a very recent Korean study using the same knowledge instrument among ICU nurses, which likewise found a high mean knowledge score (96.1%) that showed no meaningful correlation with observed practice compliance [24]; similarly, compliance with infection-prevention protocols in another recent critical-care sample was more strongly predicted by institutional and experiential factors than by knowledge alone [25]. Together, these findings suggest that ceiling-effect-driven knowledge measures may have limited discriminant and predictive value in highly trained ICU samples.

Infection control job stress was moderate (3.23/5), somewhat lower than in prior studies using the same tool (3.41–3.69) [9,14], again plausibly reflecting more standardized, less ambiguous infection control protocols after accreditation reform [22]. Quantitative workload was consistently the highest-scoring subdomain, consistent with prior research and reflecting the added physical burden of intensified infection precautions layered onto already complex ICU care [9].

Self-efficacy (3.62/5) was lower than reported among general clinical RNs using the same tool and among other nursing populations using related tools, consistent with prior evidence that ICU RNs tend to report comparatively low self-efficacy [26]. Because ICU patients’ clinical trajectories often do not improve immediately despite skilled nursing care, ICU RNs may accumulate fewer reinforcing mastery experiences, a mechanism proposed to erode self-efficacy over time even though self-efficacy is, in principle, a dynamic and modifiable resource [19,26].

Infection control performance was high overall (4.82/5), with pneumonia management the highest and hand hygiene the lowest—a pattern consistent with prior studies [18]. The comparatively low hand hygiene performance likely reflects structural constraints such as frequent emergencies and high workload rather than a lack of individual awareness [27,28].

Notably, infection control job stress was positively—not negatively—associated with infection control performance in both correlation and regression analyses, a counterintuitive pattern also reported in a minority of prior studies. This association was weak in absolute terms (r = 0.161; B = 0.065–0.073 across models, including under HC3-robust re-estimation; Section 3.6) and, given the cross-sectional design, cannot establish that job stress causes or improves performance; no nonlinear or ‘optimal-stress-level’ relationship was tested. One plausible interpretation, consistent with protection motivation theory and the Yerkes–Dodson framework, is that a moderate, work-relevant level of perceived threat may heighten vigilance [29,30]; however, this interpretation is speculative and should not be read as license for hospital management to increase workload or stressors in order to improve compliance. A methodologically simpler explanation also deserves weight: because job stress and performance were both self-reported within the same questionnaire administration, common method bias may have inflated this association, and Harman’s single-factor test (Section 2.5) cannot fully rule this out. A recent panel study similarly found that job demands were not directly associated with infection-prevention practices once safety-climate factors were accounted for [31], underscoring that the present positive association should be treated as a preliminary, hypothesis-generating finding requiring replication with objectively observed performance measures and temporally separated data collection before any practical or administrative recommendations are drawn from it.

Current department remained a significant predictor of infection control performance even after adjusting for knowledge, job stress, and self-efficacy, with surgical, emergency, trauma, general, and cardiac ICUs showing higher performance than internal medicine ICU. This is consistent with the greater concentration of invasive, procedure-related infection risk in surgical and trauma populations, where lapses in infection control are more likely to translate quickly into adverse outcomes [32], plausibly making rigorous technique more visibly and consistently reinforced in daily practice. Unstandardized coefficients and 95% confidence intervals for these department contrasts, including for the smaller cardiac-ICU (*n* = 15) and general-ICU (*n* = 20) subgroups, are reported in Table 5 (Section 3.6) to aid judgment of estimate precision.

Although statistically significant, the final regression model explained a relatively modest proportion of variance in infection control performance (17.4%), indicating a real but modest—rather than dominant—contribution of the measured knowledge and psychological factors; this distinction between statistical and practical significance is important given the large sample size. The substantial unexplained variance likely reflects unmeasured structural factors not captured in this survey, such as unit-level infection-control staffing, personal protective equipment availability, and supervisory support, as well as the restricted variance created by the ceiling effects observed in knowledge and performance scores. Consistent with this, effect sizes for the individual predictors and group comparisons in this study were generally small to medium in magnitude (Section 3.6), reinforcing that statistical significance should not be equated with strong practical importance given the sample size.

### Strengths and Limitations

This study’s strengths include a statistically justified sample size, use of previously validated instruments, and a hierarchical regression approach that clarified the incremental contribution of knowledge, job stress, and self-efficacy beyond departmental context. Several limitations should nonetheless be noted:

1. Participants were recruited by convenience sampling from two tertiary hospitals in a single province, limiting generalizability to all ICU RNs; the proportion of male RNs in this sample (15.7%) exceeded the national average among all licensed Korean RNs (approximately 7%) but was closer to the proportion among recently licensed RNs (17–18%) [33], which should be considered when generalizing findings to the broader nursing workforce. In addition, because a roster of all eligible nurses was not centrally maintained, we could not calculate a participation/response rate or compare participants with non-participants, which limits our ability to assess selection bias directly.
2. Infection control performance, job stress, and self-efficacy were all assessed by self-report within the same questionnaire administration; because these three variables were collected concurrently from a single source, CMB may have inflated or distorted their observed associations—most notably the counterintuitive positive relationship between job stress and performance—and self-reported performance may not fully reflect directly observed practice.
3. Self-efficacy was measured with a general, domain-nonspecific scale rather than an infection-control-specific self-efficacy instrument; this construct mismatch may have attenuated its association with infection control performance, and future studies should consider domain-specific self-efficacy measures.
4. The infection control knowledge tool demonstrated a KR-20 reliability coefficient of only 0.35 in this study, well below the conventional 0.70 threshold. This low coefficient is attributable to the dichotomous scoring system (correct/incorrect) of the scale combined with the extreme homogeneity of the sample (95.8% mean accuracy; Section 3.2), which sharply restricts item variance and mechanically deflates reliability estimates; item-level statistics (Section 3.6, Table 7) confirm limited discrimination for most items. Given this low reliability, the knowledge variable’s association with performance should be interpreted with substantial caution: although knowledge remained a nominally significant predictor in the original OLS regression model (β = 0.164, *p* = 0.016), this association did not survive a Spearman rank-based sensitivity analysis of the zero-order correlation, a heteroscedasticity-robust (HC3) re-estimation of the regression model, or a covariate-adjusted robustness check controlling for age, clinical/ICU experience, training, and workload (Section 3.6 and Section 3.7). We therefore regard the knowledge–performance association in this study as tentative rather than well established.
5. Both knowledge and performance scores showed a pronounced ceiling effect (95.8% and 96.4% of maximum, respectively), which restricted variance and limited the explanatory power of the hierarchical regression model (R^2^ = 0.209, adjusted R^2^ = 0.174); a substantial 79.1% of the variance remained unexplained, underscoring the likely contribution of unmeasured structural and contextual factors. The exceptionally high self-reported performance scores may also partly reflect social desirability bias, particularly given institutional pressures such as healthcare accreditation requirements, though this explanation remains speculative without corroborating observational data. Future studies should combine self-reports with objective, direct-observation methods to mitigate this bias and examine systemic organizational variables such as nurse-to-patient ratios, infection control infrastructure, and unit-level administrative support. These ceiling effects also produced non-normal, heavily left-skewed distributions for both variables (Shapiro–Wilk *p* < 0.001 for each; Section 3.2) and heteroscedastic regression residuals (Breusch–Pagan *p* < 0.001; Section 3.6). Although the independent *t*-tests, ANOVA, and OLS regression are retained for consistency with the study’s a priori analytic plan and comparability with the cited literature, and point estimates from these methods are typically reasonably robust to non-normality at this sample size, we report rank-based (Spearman) and heteroscedasticity-robust (HC3) results as sensitivity analyses (Section 3.6) and encourage readers to weigh the convergence—or, for the knowledge variable, non-convergence—of these methods when judging the strength of the evidence.
6. The cross-sectional design precludes causal inference among study variables, particularly for the job-stress–performance relationship discussed above.

## 5. Conclusions

Among ICU RNs, infection control job stress, self-efficacy, and current department were consistently associated with infection control performance across both the original and the sensitivity analyses, together with infection control knowledge explaining 17.4% of variance in the original model; however, effect sizes were small to medium, and the knowledge–performance association in particular did not survive rank-based, heteroscedasticity-robust, or covariate-adjusted sensitivity checks (Section 3.6 and Section 3.7) and should be treated as preliminary. Given the cross-sectional design, these findings should be understood as associations rather than causal or temporally ordered influences. Tentative implications include broadening infection control education beyond blood-exposure-centered content to address non-blood body-fluid precautions, and strengthening self-efficacy through hands-on training, mentoring, and supportive feedback; because this study did not test any stress-based intervention, we do not recommend deliberately sustaining or modulating ICU nurses’ job stress on the basis of these results, and any such intervention would first require experimental or longitudinal evidence. Department-tailored infection control protocols and support systems—reflecting differences in patient acuity and procedural intensity across ICU subspecialties—may further help sustain and improve infection control performance and reduce healthcare-associated infections, pending replication.

## Figures and Tables

**Table 1:** General characteristics of participants (*N* = 191).

Characteristic	Category	*n* (%)	Mean ± SD
Sex	Male Female	30 (15.7) 161 (84.3)	
Age (years)	<30 ≥30	118 (61.8) 73 (38.2)	29.73 ± 6.45
Marital status	Unmarried Married	145 (75.9) 46 (24.1)	
Education	Associate Bachelor Master or higher	31 (16.2) 151 (79.1) 9 (4.7)	
Position	Staff RN Charge RN	184 (96.3) 7 (3.7)	
Total clinical experience (years)	<3 3 to <5 5 to <10 ≥10	69 (36.1) 40 (20.9) 50 (26.2) 32 (16.8)	
Current department	Internal medicine ICU Surgical ICU Cardiac ICU Emergency ICU Trauma ICU General ICU	33 (17.3) 44 (23.0) 15 (7.9) 49 (25.7) 30 (15.7) 20 (10.5)	
ICU experience (years)	<1 1 to <3 3 to <5 ≥5	38 (19.9) 62 (32.5) 48 (25.1) 43 (22.5)	
Average daily patient load	<3 ≥3	96 (50.3) 95 (49.7)	
Infection control training sessions attended (past 12 months)	None 1 session ≥2 sessions	45 (23.6) 110 (57.6) 36 (18.8)	
Stress management methods used	None 1 method ≥2 methods	7 (3.7) 154 (80.6) 30 (15.7)	
Work shift type	3-shift 2-shift	184 (96.3) 7 (3.7)	

**Table 2:** Correlations among study variables (*N* = 191).

Variable	Knowledge, r (*p*)	Job Stress, r (*p*)	Self-Efficacy, r (*p*)	Performance, r (*p*)
Knowledge	1			
Job stress	−0.159 (0.028)	1		
Self-efficacy	0.161 (0.026)	−0.153 (0.035)	1	
Performance	0.151 (0.037)	0.161 (0.026)	0.178 (0.014)	1

**Table 3:** Hierarchical regression analysis of factors associated with infection control performance (*N* = 191).

Predictor	Model I β (*p*)	Model II β (*p*)	Model III β (*p*)	Model IV β (*p*)
Department (ref: Internal medicine ICU)				
Surgical ICU	0.421 (<0.001)	0.424 (<0.001)	0.410 (<0.001)	0.398 (<0.001)
Cardiac ICU	0.213 (0.008)	0.203 (0.010)	0.193 (0.014)	0.173 (0.026)
Emergency ICU	0.333 (<0.001)	0.327 (<0.001)	0.359 (<0.001)	0.341 (<0.001)
Trauma ICU	0.342 (<0.001)	0.347 (<0.001)	0.337 (<0.001)	0.318 (<0.001)
General ICU	0.353 (<0.001)	0.360 (<0.001)	0.337 (<0.001)	0.327 (<0.001)
Infection control knowledge	—	0.162 (0.018)	0.186 (0.007)	0.164 (0.016)
Infection control job stress	—	—	0.168 (0.019)	0.188 (0.008)
Self-efficacy	—	—	—	0.157 (0.022)

Model I: R^2^ = 0.134, Adj. R^2^ = 0.111, F = 5.742, *p* < 0.001; Model II: R^2^ = 0.160, Adj. R^2^ = 0.133, F = 5.851, *p* < 0.001; Model III: R^2^ = 0.185, Adj. R^2^ = 0.154, F = 5.947, *p* < 0.001; Model IV: R^2^ = 0.209, Adj. R^2^ = 0.174, F = 5.995, *p* < 0.001; Durbin–Watson = 1.956–1.972.

**Table 4:** Pearson and Spearman correlations among study variables, with sensitivity comparison (*N* = 191).

Pair	Pearson r	*p*	Spearman ρ	*p*
Knowledge—Job stress	−0.159	0.028	−0.149	0.040
Knowledge—Self-efficacy	0.161	0.026	0.140	0.054
Knowledge—Performance	0.151	0.037	0.034	0.636
Job stress—Self-efficacy	−0.153	0.035	−0.102	0.161
Job stress—Performance	0.161	0.026	0.190	0.009
Self-efficacy—Performance	0.178	0.014	0.175	0.016

**Table 5:** Final model (Model IV): unstandardized coefficients under conventional (OLS) and heteroscedasticity-robust (HC3) standard errors (*N* = 191).

Predictor	B	SE (OLS)	95% CI (OLS)	SE (HC3)	*p* (HC3)
Intercept	3.352	0.349	[2.663, 4.041]	0.526	<0.001
Surgical ICU (vs. internal medicine)	0.246	0.055	[0.138, 0.353]	0.067	<0.001
Cardiac ICU (vs. internal medicine)	0.167	0.074	[0.020, 0.314]	0.097	0.086
Emergency ICU (vs. internal medicine)	0.203	0.054	[0.096, 0.310]	0.071	0.005
Trauma ICU (vs. internal medicine)	0.227	0.060	[0.108, 0.345]	0.078	0.004
General ICU (vs. internal medicine)	0.277	0.068	[0.144, 0.411]	0.070	<0.001
Infection control knowledge	0.040	0.017	[0.008, 0.073]	0.024	0.095
Infection control job stress	0.073	0.027	[0.019, 0.127]	0.026	0.005
Self-efficacy	0.077	0.033	[0.011, 0.142]	0.033	0.021

**Table 6:** Effect sizes with 95% confidence intervals for significant group comparisons (Section 3.3).

Comparison	Test Statistic	*p*	Effect Size	95% CI
Sex → Job stress	Welch t = −3.31	0.002	Cohen’s d = −0.69	[−1.08, −0.29]
Marital status → Job stress	Welch t = −2.71	0.008	Cohen’s d = −0.43	[−0.77, −0.10]
Age (<30/≥30) → Job stress	Welch t = −2.87	0.005	Cohen’s d = −0.43	[−0.73, −0.14]
Education → Job stress	F(2, 188) = 5.29	0.006	η^2^ = 0.053	[0.010, 0.145]
Clinical experience → Job stress	F(3, 187) = 4.87	0.003	η^2^ = 0.073	[0.027, 0.169]
Clinical experience → Knowledge	F(3, 187) = 2.85	0.039	η^2^ = 0.044	[0.011, 0.127]
Department → Job stress	F(5, 185) = 3.64	0.004	η^2^ = 0.090	[0.046, 0.201]
Department → Performance	Welch F(5, 67.1) = 4.48	0.001	η^2^ = 0.134	[0.068, 0.264]
ICU experience → Job stress	F(3, 187) = 2.71	0.047	η^2^ = 0.042	[0.010, 0.127]
Training sessions → Self-efficacy	F(2, 188) = 3.22	0.042	η^2^ = 0.033	[0.005, 0.110]

**Table 7:** Item-level statistics for the 20-item infection control knowledge scale (*N* = 191).

Item	% Correct	Item-Total r (Corrected)	*p*
4	84.3	0.312	<0.001
2	84.8	0.236	0.001
17	88.5	0.134	0.064
20	92.7	−0.015	0.837
6	94.2	0.119	0.101
8	95.8	0.065	0.368
13	96.3	0.260	<0.001
18	96.3	0.036	0.619
19	96.9	0.032	0.656
12	96.9	0.150	0.038
15	96.9	0.091	0.212
1	97.9	−0.010	0.891
7	98.4	−0.058	0.422
3	99.0	−0.032	0.659
9	99.0	−0.080	0.271
5	99.5	0.080	0.273
11	99.5	0.219	0.002
16	99.5	−0.057	0.434
14	99.5	0.149	0.040
10	100.0	n/a (constant)	n/a

**Table 8:** Covariate-adjusted robustness check (Model V): conventional (OLS) and heteroscedasticity-robust (HC3) estimates (*N* = 191).

Predictor	B	SE (OLS)	*p* (OLS)	SE (HC3)	*p* (HC3)	VIF
(Intercept)	3.472	0.364	<0.001	0.503	<0.001	—
Surgical ICU (vs. IM ICU)	0.237	0.055	<0.001	0.066	<0.001	1.87
Cardiac ICU (vs. IM ICU)	0.157	0.076	0.041	0.096	0.103	1.42
Emergency ICU (vs. IM ICU)	0.192	0.054	0.001	0.072	0.008	1.93
Trauma ICU (vs. IM ICU)	0.223	0.061	<0.001	0.078	0.005	1.68
General ICU (vs. IM ICU)	0.275	0.070	<0.001	0.072	<0.001	1.55
Infection control knowledge	0.041	0.017	0.015	0.024	0.091	1.10
Infection control job stress	0.078	0.028	0.005	0.027	0.004	1.18
Self-efficacy	0.086	0.034	0.014	0.035	0.015	1.15
Age (years)	−0.007	0.005	0.158	0.006	0.271	3.17
Total clinical experience (ordinal, 1–4)	0.013	0.031	0.667	0.039	0.734	3.95
ICU experience (ordinal, 1–4)	0.007	0.019	0.695	0.019	0.698	1.33
Training sessions (ordinal, 0/1/≥2)	−0.033	0.028	0.229	0.031	0.284	1.10
Average daily patient load (≥3 vs. <3)	−0.009	0.037	0.809	0.036	0.802	1.19

IM ICU = internal medicine ICU (reference category). HC3 = heteroscedasticity-consistent (HC3) robust standard errors. VIF = variance inflation factor (from the OLS model).

## Data Availability

The datasets generated and analyzed during the current study are not publicly available due to privacy and ethical restrictions imposed by the Institutional Review Board of Wonkwang University, as participants did not consent to public sharing of their raw data. Minimal anonymized data may be made available from the corresponding author upon reasonable request and with permission of the participating hospitals.

## Supplementary Materials

The following supporting information can be downloaded at: https://www.mdpi.com/article/10.3390/healthcare14203443/s1, Table S1: STROBE Statement—checklist of items that should be included in reports of cross-sectional studies.
