# Supplementary material for: Infection Control Knowledge, Job Stress, and Self-Efficacy in Relation to Infection Control Performance Among Intensive Care Unit Registered Nurses: A Descriptive Cross-Sectional Study
Source: Healthcare (Basel). 2026 Oct 14;14(20):3443. doi: 10.3390/healthcare14203443 (PMC13704700; doi:10.3390/healthcare14203443)
Supplement: Supplementary file 1 [file healthcare-14-03443-s001.zip › healthcare-4514034-supplementary.pdf]

Supplementary Materials

Infection Control Knowledge, Job Stress, and Self-Efficacy in Relation to Infection Control Performance Among Intensive Care Unit Registered Nurses: A Descriptive Cross-Sectional Study

Ji Soo Lee [1] and Eun Young Jeong [2,*]

[1] Department of Nursing, Graduate School, Wonkwang University, Iksan 54538, Republic of Korea

[2] College of Nursing, Wonkwang University, Iksan 54538, Republic of Korea

* Correspondence: pionaorai65@wku.ac.kr

Table S1. STROBE Statement—Checklist of items that should be included in reports of cross-sectional studies.

Item No.	Recommendation	Reported in (Section/Table)
**TITLE and ABSTRACT**		
**1**	(a) Indicate the study's design with a commonly used term in the title or the abstract	Title; Abstract (Methods)
	(b) Provide in the abstract an informative and balanced summary of what was done and what was found	Abstract
**INTRODUCTION**		
**2: Background/rationale**	Explain the scientific background and rationale for the investigation being reported	Section 1 (Introduction)
**3: Objectives**	State specific objectives, including any prespecified hypotheses	Section 1 (Introduction; study aims)
**METHODS**		
**4: Study design**	Present key elements of study design early in the paper	Section 2.1 (Study Design)
**5: Setting**	Describe the setting, locations, and relevant dates, including periods of recruitment, exposure, follow-up, and data collection	Section 2.2 (Participants); Section 2.4 (Data Collection)
**6: Participants**	Give the eligibility criteria, and the sources and methods of selection of participants	Section 2.2 (Participants)
**7: Variables**	Clearly define all outcomes, exposures, predictors, potential confounders, and effect modifiers. Give diagnostic criteria, if applicable	Section 2.3 (Measures)
**8: Data sources/ measurement**	For each variable of interest, give sources of data and details of methods of assessment (measurement). Describe	Section 2.3 (Measures)

	comparability of assessment methods if there is more than one group	
**9: Bias**	Describe any efforts to address potential sources of bias	Section 2.2 (exclusion of incomplete/insincere responses); Section 2.5 (common method bias; multiple comparisons); Section 4 (Strengths and Limitations)
**10: Study size**	Explain how the study size was arrived at	Section 2.2 (Participants; G*Power calculation)
**11: Quantitative variables**	Explain how quantitative variables were handled in the analyses. If applicable, describe which groupings were chosen and why	Section 2.5 (Statistical Analysis)
**12: Statistical methods**	(a) Describe all statistical methods, including those used to control for confounding	Section 2.5 (Statistical Analysis)
	(b) Describe any methods used to examine subgroups and interactions	Section 2.5 (Statistical Analysis)
	(c) Explain how missing data were addressed	Section 2.2 (Participants; 28 questionnaires excluded for incomplete [n = 25] or insincere [n = 3] responses)
	(d) If applicable, describe analytical methods taking account of sampling strategy	N/A (convenience sample; sampling-strategy-adjusted analysis not applicable)
	(e) Describe any sensitivity analyses	Section 2.5 (Statistical Analysis); Section 3.6 (Spearman correlations, residual diagnostics, and HC3 robust standard errors; Tables 4 and 5); Section 3.7 (covariate-adjusted robustness check; Table 8)
**RESULTS**		
**13: Participants**	(a) Report numbers of individuals at each stage of study—eg numbers potentially eligible, examined for eligibility, confirmed eligible, included in the study, completing follow-up, and analysed	Section 2.2 (Participants)
	(b) Give reasons for non-participation at each stage	Section 2.2 (Participants)
	(c) Consider use of a flow diagram	Not applicable — a flow diagram was not included; participant flow (n = 219 recruited; n = 28 excluded for incomplete/insincere responses; n = 191 analyzed) is reported narratively in Section 2.2 (Participants).
**14: Descriptive data**	(a) Give characteristics of study participants (eg demographic, clinical, social) and information on exposures and potential confounders	Section 3.1 (General Characteristics of Participants; Table 1)

	(b) Indicate number of participants with missing data for each variable of interest	Section 2.2 (Participants)
**15: Outcome data**	Report numbers of outcome events or summary measures	Section 3.2 (Levels of Infection Control Knowledge, Job Stress, Self-Efficacy, and Performance)
**16: Main results**	(a) Give unadjusted estimates and, if applicable, confounder-adjusted estimates and their precision (eg, 95% confidence interval). Make clear which confounders were adjusted for and why they were included	Section 3.4 (unadjusted correlations; Table 2); Section 3.5 (Factors Associated with Infection Control Performance; Table 3); Section 3.6 (unstandardized coefficients with 95% CIs; Table 5); Section 3.7 (covariate-adjusted estimates; Table 8)
	(b) Report category boundaries when continuous variables were categorized	Section 3.1 (Table 1)
	(c) If relevant, consider translating estimates of relative risk into absolute risk for a meaningful time period	N/A (continuous outcome; relative-risk translation not applicable)
**17: Other analyses**	Report other analyses done—eg analyses of subgroups and interactions, and sensitivity analyses	Section 3.3 (Differences in Study Variables by General Characteristics); Section 3.4 (Correlations Among Study Variables; Table 2); Section 3.6 (sensitivity and diagnostic analyses; Tables 4–7); Section 3.7 (covariate-adjusted robustness check; Table 8)
**DISCUSSION**		
**18: Key results**	Summarize key results with reference to study objectives	Section 4 (Discussion, first paragraph)
**19: Limitations**	Discuss limitations of the study, taking into account sources of potential bias or imprecision. Discuss both direction and magnitude of any potential bias	Section 4 (Strengths and Limitations)
**20: Interpretation**	Give a cautious overall interpretation of results considering objectives, limitations, multiplicity of analyses, results from similar studies, and other relevant evidence	Section 4 (Discussion); Section 5 (Conclusions)
**21: Generalisability**	Discuss the generalisability (external validity) of the study results	Section 4 (Strengths and Limitations; sampling and sex-distribution discussion)
**OTHER INFORMATION**		
**22: Funding**	Give the source of funding and the role of the funders for the present study and, if applicable, for the original study on which the present article is based	Back Matter (Funding)
